# Leveraging natural capital accounting to support businesses with nature-related risk assessments and disclosures

**DOI:** 10.1098/rstb.2022.0328

**Published:** 2024-06-10

**Authors:** Jane Carter Ingram, Emily J. McKenzie, Kenneth J. Bagstad, John Finisdore, Rayne van den Berg, Eli Fenichel, Michael Vardon, Stephen Posner, Marta Santamaria, Lisa Mandle, Richard Barker, James Spurgeon

**Affiliations:** ^1^ Pollination, Washington, DC 20037, USA; ^2^ Taskforce on Nature-related Financial Disclosures, London EC1A 2BN, UK; ^3^ United States Geological Survey, Reston, VA 20192, USA; ^4^ Point Advisory, ERM, Melbourne, VIC 3000, Australia; ^5^ Forico, Kings Meadows, TAS 7249, Australia; ^6^ School of Forestry and Environmental Studies Studies, Yale University, New Haven, CT 06511, USA; ^7^ Australian National University, Canberra, ACT 2601, Australia; ^8^ Garrison Institute, Garrison, NY 10524, USA; ^9^ Capitals Coalition, London EC2R 6JH, UK; ^10^ Stanford University, Stanford, CA 94305, USA; ^11^ International Financial Reporting Standards, London E14 4HD, UK; ^12^ Sustain Value, London, London SW19 2RR, UK

**Keywords:** natural capital accounts, nature risk, system of environmental-economic accounting, business, Taskforce on Nature-related Financial Disclosures

## Abstract

Nature loss threatens businesses, the global economy and financial stability. Understanding and addressing these risks for business will require credible measurement approaches and data. This paper explores how natural capital accounting (NCA) can support business data and information needs related to nature, including disclosures aligned with the Taskforce on Nature-related Financial Disclosures recommendations. As businesses seek to measure, manage and disclose their nature-related risks and opportunities, they will need well-organized, consistent and high-quality information regarding their dependencies and impacts on nature, which few businesses currently collect or track in-house. NCA may be useful for these purposes but has not been widely used or applied by businesses. National NCA guided by the U.N. System of Environmental-Economic Accounting may provide: (i) a useful framework for businesses in conceptualizing, organizing and managing nature-related data and statistics; and (ii) data and information that can directly support business disclosures, corporate NCA and other business applications. This paper explores these opportunities as well as synergies between national and corporate natural capital accounts. In addition, the paper discusses key barriers to advancing the wider use and benefits of NCA for business, including: awareness of NCA, data access, business capabilities related to NCA, spatial and temporal scales of data, audit and assurance considerations, potential risks, and costs and incentives.

This article is part of the theme issue ‘Bringing nature into decision-making’.

## 1. Introduction

The world is losing nature at unprecedented rates [1]. Since natural capital underpins the global economy, nature degradation reduces opportunities for future economic production and diminishes human well-being [2]. A recent study estimated that approximately US $44 trillion of economic value generation is highly or moderately dependent on nature [3]. Industries that are vulnerable to nature loss may need to proactively manage these nature-related risks to avoid negative business implications and may decide to positively impact nature in ways that generate new business opportunities [3]. However, the majority of businesses, investors and lenders are not yet aware of, nor measuring or monitoring, their nature-related dependencies and impacts, and how these may lead to financial risks and opportunities across supply chains, operations, loan books or investment portfolios [4]. For example, the World Benchmarking Alliance [5] reported that out of approximately 400 companies analysed in a recent study, only 5% have measured their impacts on nature and just 1% know their dependencies on nature. Such findings could represent areas of potential concern, particularly since dependencies on nature can be critical for business operations and sourcing.

One challenge to business leaders' efforts to manage dependencies and impacts on nature is that they frequently lack access to well-organized, high-quality, credible and relevant data [6] and the internal capacity to process, interpret and apply relevant data for informing business and investor decisions. The growing recognition of the importance that businesses understand and disclose nature-related issues is evidenced by Target 15 of the 2022 Kunming–Montreal Global Biodiversity Framework, which states that parties to the Convention on Biological Diversity should take measures to encourage and enable large and transnational companies and financial institutions to ‘regularly monitor, assess and transparently disclose their risks, dependencies and impacts on biodiversity’ [7]. The principle underpinning Target 15 was supported by over 300 businesses in the build-up to the negotiations on the Global Biodiversity Framework [8]. Businesses are increasingly aware of the value of nature, the physical and transition risks that nature loss creates for businesses, and growing pressures from investors and regulators to disclose their nature-related risks. In response to emerging frameworks such as the Taskforce on Nature-related Financial Disclosures (TNFD) recommendations and emerging regulatory standards such as the European Sustainability Reporting Standards, business-relevant natural capital tools and data have proliferated [9].

Despite this proliferation in data and tools, few companies continuously, consistently and systematically measure and manage business-relevant dependencies and/or impacts on nature using natural capital accounting (NCA) to collect and organize data. Although the concept of corporate NCA (CNCA) is emerging [10] and a growing number of companies are adopting CNCA, its use to inform business decisions is not yet common practice.

By contrast, 92 countries are now developing national natural capital accounts (NNCA) aligned with the System of Environmental-Economic Accounting (SEEA) at a country-wide level to increase the integration of the values of nature into decision-making [11]. While NNCA are typically compiled at the national scale, they are by design multi-scale, with results often reported for subnational political and/or biophysical units (e.g. at the level of watersheds or ecoregions). Additionally, NNCA, particularly ecosystem accounts, typically include spatially explicit data at fine to moderate resolution, which can be re-aggregated by users to any desired custom geography. This enables NNCA data to be used directly by corporations or other entities at local levels where nature-related impacts and dependencies manifest, and could make NNCA data highly useful to corporations and other users in similar ways in which the data and tools of national economic accounting have proven useful. National-level commitments to implement NCA provide critical signals that governments recognize the values of nature and that it should be integrated into economic decisions and planning. Australia, Botswana, Brazil, Canada, Colombia, Indonesia, Mexico, the Netherlands, South Africa, the United Kingdom and others have implemented NNCA aligned with the SEEA Central Framework and SEEA Ecosystem Accounts [11]. Notably, the United States released a national strategy on NCA in January 2023 [12].

This paper explores the roles that NCA can play in supporting business strategies and decisions related to assessing, managing and reporting on nature-related risks and opportunities, including in alignment with the Taskforce on Nature-related Financial Disclosures (TNFD). In particular, we address how NCA can support businesses by providing a structure to organize and manage high-quality and credible data related to nature, including physical and monetary measures of natural capital stocks and ecosystem service flows relevant to impacts and dependencies beyond the direct control of business and which they may not track on their own. We aim to advance awareness of the value and benefits of NCA within the business community and opportunities for cooperation and integration among businesses and governmental NCA efforts, using TNFD-aligned disclosures as a tangible example of a business application that could benefit from NCA data and information. Increasing business access to and use of credible nature data to inform decisions and risk management may also help businesses sustainably use, manage and positively impact nature in ways that benefit biodiversity and society and support business outcomes. In addition, greater collaboration between business and government-led NCA efforts may help drive the awareness of, use and application of environmental data in economic and financial decision-making. Because many NNCA efforts are so new, effective communication and collaboration between the public and private sectors can ensure the development of accounts that are needed for decision-making and to build a ‘policy pull’ for relevant NCA information [13,14].

The paper is structured as follows. First, we describe the rapidly emerging focus on natural capital within the business community, including the recent development of the TNFD. Second, we detail the current state of NNCA and CNCA, and potential synergies between them. Third, we illustrate these potential synergies and the benefits of NCA for businesses through a case study describing the CNCA efforts of Forico, an Australian forestry company. Fourth, we describe how NNCA and CNCA could interact and mutually reinforce the development and application of both approaches to NCA. Fifth, we discuss critical barriers and steps that need to be addressed to encourage the wider adoption of NCA by businesses.

## 2. Emerging business needs for natural capital data and information: the TNFD framework

Increasingly, businesses are being asked to measure and disclose their nature-related dependencies, impacts, risks and opportunities in response to expectations set out in the 2022 Global Biodiversity Framework, recent statements by policy-makers such as the G7 Alliance for Nature-Positive Economies [15], evolving voluntary and mandatory standards for nature reporting, increasing shareholder activism and investor requests related to nature risks [7,16–20]. For example, in cases in which natural capital is material to the long-term strategy of companies, large institutional investors such as Blackrock are requesting corporate disclosures that include assessments of risk, risk oversight and understanding of how dependencies and impacts on nature are managed [17]. Target 15 of the 2022 Global Biodiversity Framework specifies that governments should enable and encourage businesses to regularly monitor, assess and transparently disclose their risks, dependencies and impacts on biodiversity throughout their supply chains, operations and portfolios [7]. Certain jurisdictions are creating initiatives and legislation on corporate disclosures on nature. For example, the European Union (EU) regulation on deforestation-free supply chains will require companies to conduct strict due diligence related to deforestation and degradation impacts of commodities that they place on the EU market [19]. The European Sustainability Reporting Standards developed by the European Financial Reporting Advisory Group set out mandatory reporting standards for nature-related issues, following the European Corporate Sustainability Reporting Directive [18]. In addition, in June 2023, the International Sustainability Standards Board published its inaugural global sustainability disclosure standards [21], and in December 2022 announced that it will research enhancements that complement the Climate-related Disclosures Standard, relating to natural ecosystems [21]. Market regulators in other jurisdictions are also developing nature-related standards and reporting requirements, such as the Securities and Exchange Board of India's Business Responsibility and Sustainability Reporting standards. In addition, in 2023, the New York Stock Exchange (NYSE) submitted a proposal to the U.S. Securities and Exchange Commission to amend the NYSE Listed Company Manual to adopt an additional listing standard for a new type of public company referred to as Natural Asset Companies [22]. Under the proposal rules, Natural Asset Companies would conduct sustainable revenue-generating operations and would not have adverse impacts on nature. The proposal would also require these companies to measure, value and report on ecosystem services and natural assets that they manage and impact using a reporting framework aligned to the U.N. SEEA Ecosystem Accounting [22]. However, the proposal was withdrawn by the NYSE in January 2024.

The TNFD is a market-led taskforce that has developed a risk management and disclosure framework for businesses on nature-related risks and opportunities, with a set of recommended disclosures and supporting guidance [4]. The TNFD framework builds on and aligns to other corporate nature frameworks and methodologies including SEEA Ecosystem Accounting, and other frameworks such as the Natural Capital Protocol (for natural capital assessments and valuation) and the Science Based Targets Network's guidance on setting science-based targets for nature [23].

**Figure 1.**
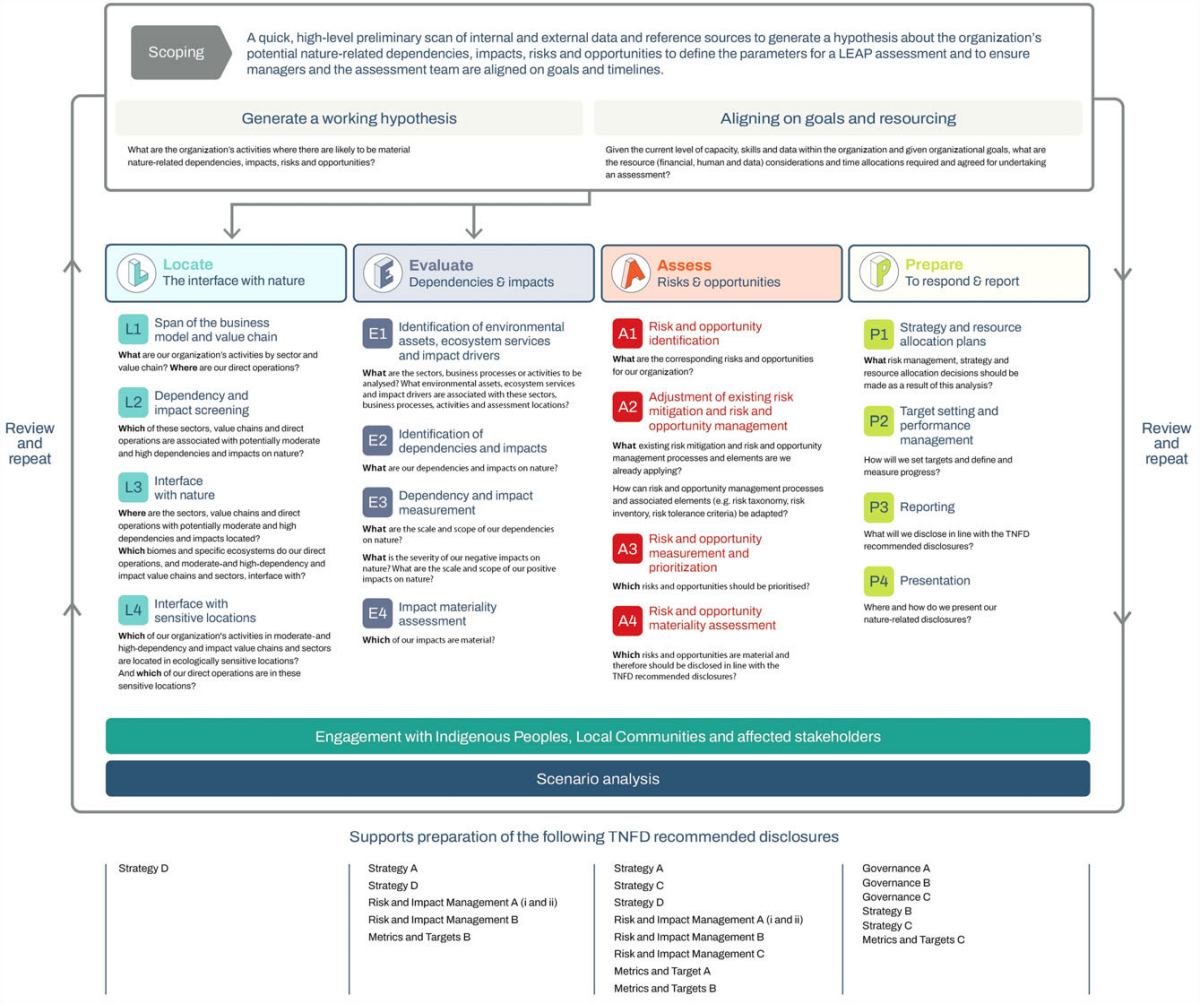
The locate, evaluate, assess, prepare (LEAP) approach for identification and assessment of nature-related issues [25].

The TNFD-recommended disclosures follow the structure of the Task Force on Climate-related Financial Disclosures (TCFD) recommendations on governance, strategy, risk management and metrics and targets. Approximately 15 countries have made TCFD disclosures mandatory [24] and the TCFD's recommendations are now folded into the global sustainability reporting baseline through the International Sustainability Standards Board's Climate-related Disclosures Standard. One key new element of the TNFD framework is recognition that, unlike greenhouse gas emissions and removals, nature-related dependencies and impacts are location-specific. Thus, to understand nature-related risks and opportunities related to supply chains and/or operations, the TNFD recommends that businesses collect and use data and information on nature at local levels. To do so, TNFD proposes an approach referred to as 'Locate, Evaluate, Assess and Prepare' (LEAP), where a business (i) locates its interfaces with nature, for instance by identifying the biomes and ecosystems with which its operations interact; (ii) evaluates its dependencies and impacts on nature, e.g. in terms of species, ecosystem extent and condition, and ecosystem services in specific locations; (iii) assesses material risks and opportunities relevant to its dependencies and impacts; and 4) prepares to respond to and report on these issues ([25]; figure 1). Many, if not most, businesses are unlikely to have all of this information in-house, especially at adequately high-spatial resolutions and/or at landscape scales, at which most nature-related impacts and dependencies manifest. Thus, many businesses will need to rely on third-party data and tools to support these analyses [9].

Because of the growth in emerging public and private drivers related to business disclosures on nature, it is timely to explore how government-led national and/or corporate-led NCA can support businesses with the data, structure and concepts for organizing and managing the nature-related information needed for business applications such as TNFD reporting.

## 3. Natural capital accounting as a way to structure and manage information for businesses

NCA refers to the use of a structured, rules-based framework to systematically measure and report on stocks of natural capital and flows of ecosystem services. The practice of NCA has grown in both the private and public sectors worldwide in recent years. Both are similar with respect to concepts and organizing structures for tracking natural capital data and information. However, the comprehensiveness of the natural capital stocks and ecosystem services addressed and spatial and temporal scales of the data may differ across NNCA and CNCA. Both can be useful for supporting business decisions related to investment opportunities, risk management, sourcing strategies and stakeholder engagement.

### (a) Government-led national NCA

SEEA is the accepted international standard for NCA and provides a framework for organizing and presenting statistics on the environment and its relationship with the economy. SEEA includes two components: the Central Framework, established as a statistical standard in 2012, and Ecosystem Accounting, which dates to 2021 [26]. The SEEA Ecosystem Accounting chapters describing its background and physical accounts were elevated to a statistical standard, while those addressing valuation were labelled as ‘statistical principles and recommendations' requiring further research and application to develop robust, national-scale valuation approaches compatible with the System of National Accounts [27,28]. The SEEA Central Framework and Ecosystem Accounting statistical standards solidify the concepts for NCA and provide definitions, accounting treatments, table structures and guidance on the information types needed for accounts.

The SEEA provides information in physical and monetary terms related to environmental stocks and flows between the environment and the economy. SEEA is capable of addressing multiple thematic areas including agriculture, forests and fisheries, energy, greenhouse gas emissions, environmental activities (expenditures, taxes and subsidies) and land, water and ecosystem accounts, the last of which considers ecosystems and how individual environmental assets interact as part of natural processes within a given spatial area [26].

Ninety-two countries are now developing NCA that are using the SEEA to strengthen the incorporation of nature into decision-making and policies [11]. SEEA's coverage, structure and value concepts also have informed the structure of CNCA [10] because SEEA's standards, transparency and rigour meet many of the data and information standards important for businesses [6,14]. Reflecting this, the TNFD has incorporated the SEEA categories and definitions of environmental and ecosystem assets, ecosystem services and biomes into its core concepts [4,25].

### (b) Corporate natural capital accounting

In addition to government-led national NCA, CNCA can be developed and managed by individual businesses to inform nature-related decisions. The use of CNCA is growing alongside numerous approaches to measuring and reporting business interactions with nature that have evolved over recent years [10,29]. The Natural Capital Protocol emerged in 2016 as a harmonized framework of different methods for corporate natural capital assessments and valuation (Capitals Coalition [30]). Other initiatives such as Align^1^ and Transparent^2^ have sought to further advance corporate approaches to natural capital measurement, valuation and accounting. In this paper, we rely on the CNCA definition and structure provided by the Capitals Coalition that has been informed by these previous efforts, as well as the Biological Diversity Protocol [31], SEEA [26] and the BS8632 Standard on Natural Capital Accounting for Organizations [32], and is defined as:

‘the systematic process of identifying, measuring, recording, summarising and reporting the periodic and accumulated net changes to (a) the biophysical state of natural capital assets and (b) the associated values of natural capital to business and wider society’ [10].

**Figure 2.**
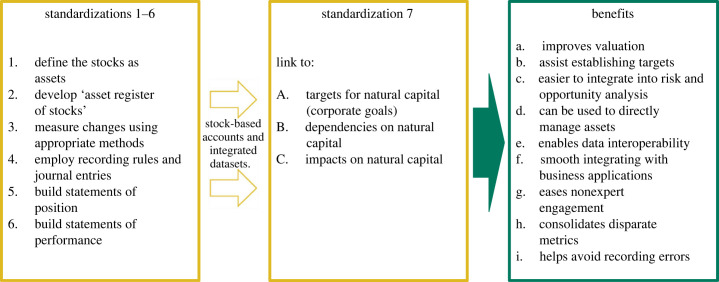
Seven steps or ‘standardizations’ associated with CNCA can support nine potential benefits to businesses. Source: [10].

A key organizational aspect of CNCA lies in its definition provided by the Capitals Coalition, specifying that CNCA follow seven ‘standardizations’ (akin to steps or accounting rules) (figure 2) that need to be implemented sequentially in order to realize complete accounts and integrated datasets that organize biophysical data using methods from financial accounting.^3^ While the emphasis of these standardizations differs somewhat from that of NNCA aligned to SEEA (for instance, the spatial units of greatest relevance to national governments and corporations differ), they are completely compatible with SEEA's structure and accounting rules. Using this approach, CNCA can support numerous business applications from the production of robust, repeatable and auditable natural capital data that may be compared among sites, business units and across different businesses, and used for developing targets, measuring dependencies and impacts, valuation and disclosures among other business applications [10].

A growing number of businesses are using CNCA, such as BHP [34], which recently published their first CNCA, and Forico [35], which has published CNCA for the years 2020–2022. CNCA may be easiest for primary industries (e.g. mining, forestry, agriculture) to implement, as many of these businesses already collect and manage some of the relevant data and have direct interest in gaining more insights from these datasets. To expand uptake of CNCA for a wider range of sectors and businesses, several challenges need to be addressed including consistency in methods, digitalization and data flows, and knowledge and skills gaps [10]. Opportunities and pathways to address some of these challenges as they pertain to both government-led NNCA and CNCA are outlined in box 1.

Box 1. **Advancing a global data platform for NCA data.**

Multi-national businesses benefit from national economic accounts data, which are built on the common System of National Accounts that dates to the 1950s, was updated in 2008 and is scheduled for a new update in 2025 [36]. System of National Accounts data are summarized into common databases by major international organizations such as the World Bank, International Monetary Fund and the Organization for Economic Co-operation and Development. By contrast, the SEEA Central Framework and Ecosystem Accounting are relatively new. While countries are scaling up and still experimenting with the content of their SEEA accounts, the few international databases that do exist (e.g. the World Bank's Changing Wealth of Nations [37]) are incomplete. For a business operating in a dozen countries, NNCA data may likely be partial and inconsistent in the near term. Large data gaps are common in many nations, particularly in the Global South, where many agricultural products and raw materials are sourced and where nature loss rates are high. However, for businesses that need to access data on the extent and condition of land, water and ecosystems in places relevant to their direct operations and/or upstream and downstream value chains to support TNFD-aligned analyses and reporting, NNCA has the potential to provide useful information, particularly as more countries develop and advance SEEA accounts that are complete enough to provide the data that businesses need.

Ideally, global NCA databases would provide well-organized, reliable and trusted data, including spatial data, that businesses could easily access to integrate into CNCA and/or use directly for business applications, including but not limited to TNFD, for locations anywhere in the world. This would require a global standard for data to be considered credible for NCA purposes, since many derived, model-generated data products exist, but not all are suitable for NCA. Global data could be replaced by national NCA data and, potentially, regional data where available, while the relative quality and compatibility of global, national and subnational NCA products would be noted. A global NCA database or data facility would require the development and use of global classification systems over country or regional classification systems; existing global economic accounts databases already address this issue.

Several existing initiatives could bridge the gap between today's status quo and a future that includes reliable global NCA databases or data facilities that provide data for use by businesses. First, the U.N. Global Platform [38] is a cloud-based collaboration environment for the global statistical community. It provides a venue for countries to share geospatial data products, including NCA data, and could be used to ensure the use of common metadata and geospatial data standards (e.g. cloud-optimized file formats that enable automated data access) for contributed data products. Second, TNFD has proposed the creation of a Global Nature-Related Public Data Facility [39], which would ideally maintain compatibility with the U.N. Global Platform. Third, Artificial Intelligence for Environment and Sustainability (ARIES) for SEEA is already used to support NNCA. ARIES for SEEA enables automated compilation of accounts that integrate global and national NCA data, which have been made discoverable and accessible using carefully applied semantics (i.e. metadata descriptors) and provenance that enable accurate substitution of higher-quality national or subnational products to replace global ones, where they are available [40]. This approach addresses the three critical challenges to applying CNCA noted by the Capitals Coalition—(i) consistency in methods (ii) digitalization and data flows and (iii) knowledge and skills gaps—addressed, respectively, by rigorous semantics, a global interoperable platform for NCA data across geographies and artificial intelligence assistance to navigate and assemble best-available NCA data while transparently reporting on their provenance.

## 4. The use of NCA to inform TNFD disclosures: the Forico example

**Figure 3.**
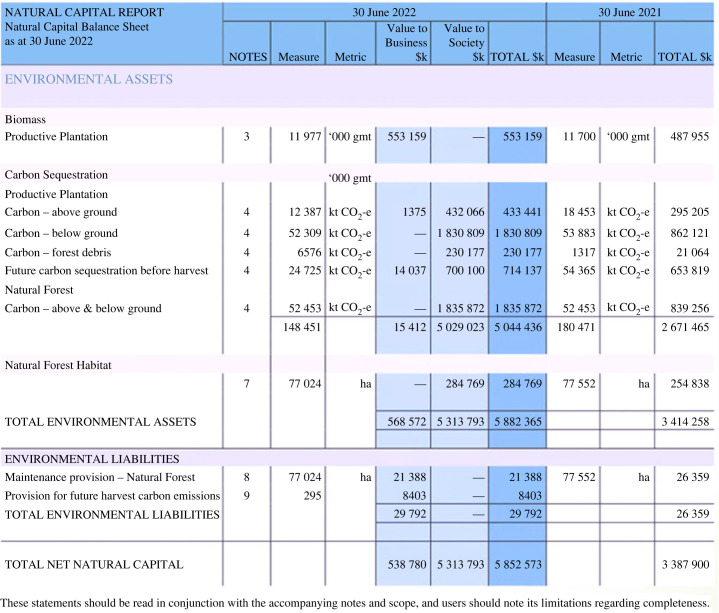
The Natural Capital Balance Sheet presented in Forico's Natural Capital Report. The balance sheet shows the assets and liabilities from natural capital stocks or ecosystem services valued over the remaining planned lifecycle of the assets managed by Forico on 30 June 2022. Only the assets that were able to be measured and assigned a monetary value with a reasonable level of confidence were included, which meant some ecosystem benefits that are important for the company are not included at this time (e.g. air filtration, soil and nutrient regulation, and flood mitigation). All monetary figures are presented in Australian Dollars. Source: [35].

One example of a business regularly publishing NCA and using the integrated datasets they provide to support TNFD reporting is Forico, a forestry-based company based in Tasmania. Forico is Tasmania's largest private forestry management company, providing wood fibres and managing approximately 173 000 hectares of land. Forico has developed NCAs for the financial years ending 30 June 2020, 2021 and 2022, and intends to update their accounts every year in alignment with existing financial reporting cycles [35,41,42]. Through this process, they have demonstrated the considerable value that is generated from the forests they manage, in addition to what they generate from timber-related products. Their NCA reports claim that their contribution to other ecosystem services exceeds the value of timber-related products [43]. As shown in figure 3, Forico focused on measuring and valuing provisioning services (wood fibre from sustainable plantations to be converted to sawlogs and wood fibre products) and regulating services (carbon sequestration from plantation and natural forests, water usage and impact on both high and low downstream flows, control of erosion and sediment retention by riparian areas and habitat areas for supporting biodiversity) and impacts from carbon emissions from supply chains using methods and approaches described in their latest Natural Capital Report [43]. In some cases, such as water regulation, services were assessed using publicly available government data and models, demonstrating the potential for alignment between government-led and business approaches to natural capital measurement.

In their report, Forico acknowledges that while standardization of NCA is an emerging field, they fully disclose their measurement and valuation methodologies so users can make their own assessments as to the materiality and rigour of their published quantitative metrics and disclosures. In addition, Forico notes that their approach to NCA continues to evolve. Their early efforts to measure and record environmental benefits from their estate (from 2016 to 2019) applied the concepts and framework of the SEEA Ecosystem Accounting [44]. In their most recent Natural Capital report, they have been guided by fundamental accounting concepts in determining the criteria for assets, liabilities, expenses and revenue as per the definitions provided by the Australian Accounting Standards Board (AASB) Framework [35]. Although government-led NCA exists and continues to be advanced in Australia, Forico acknowledges the current absence of applicable Australian or international accounting standards for CNCA [35]. As Forico continues to expand their data, SEEA-informed CNCA standards continue to develop, materiality assessments change with evolving external factors and markets begin to value the broader range of services provided by forests, the business intends to advance their accounts to include more ecosystem services and present a more comprehensive picture of natural capital values that they impact and depend upon. Forico also has published an integrated TCFD and TNFD report using data and information from their NCA and Strategic Planning assessments [43]. Their NCA work has been core to accessing data and information needed to report materiality, dependencies and impacts on nature, nature-related risks and opportunities for the organization, and metrics associated with both of these disclosure frameworks.

**Figure 4.**
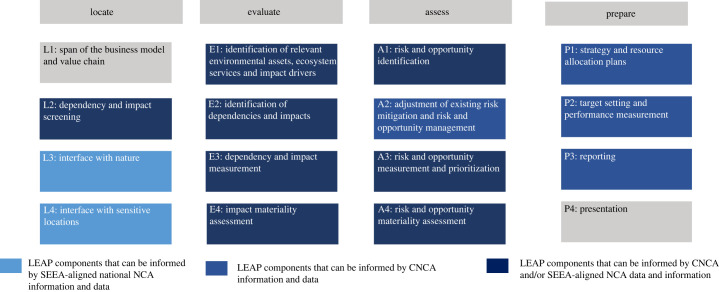
Illustration of the potential for national natural capital accounting (NNCA) and corporate natural capital accounting (CNCA) data and information to be applied to evaluate and assess nature-related business dependencies, impacts, risks and opportunities in alignment with the components of the TNFD locate, evaluate, assess, prepare (LEAP) approach [4,25,39]. Note: this is for illustration purposes only and based on the data and information that existing examples of System of Environmental-Economic Accounting (SEEA)-aligned NNCAs and CNCAs have compiled, organized and tracked consistently over time.

Forico has also demonstrated how NNCA and/or CNCA data can provide location-specific data on nature risks and opportunities that are useful for following TNFD's voluntary LEAP approach [25,43]:

— In the Locate (L) phase, CNCA and NNCA data related to ecosystem extent and condition can help companies to screen their potentially moderate and high dependencies and impacts on nature associated with their direct operations (component L2). NNCA can be used to identify the relevant biomes and ecosystems with which the business is interfacing in the locations of its direct operations and activities in its upstream and downstream value chain(s) (L3). NNCA can be especially useful for the prioritization of ecologically sensitive locations for business assets and activities, avoiding for instance areas of high ecosystem integrity, rapid decline in ecosystem integrity, high physical water risks, or importance for biodiversity or ecosystem service provision (L4).
— Throughout all components of the Evaluate phase, CNCA and NNCA can provide useful information to identify relevant environmental assets and ecosystem services, and can enable identification and measurement of a business's priority dependencies and impacts on nature, based on an assessment of impact drivers, the state of nature and provision of ecosystem services.
— In the Assess phase (A), CNCA and NNCA can provide useful information in components A1, A3 and A4 by providing data needed to assess the material risks and opportunities to a business based on their exposure to dependencies and impacts on nature. CNCA can also inform the identification of current and additional nature-related risk management efforts (A2).
— In the Prepare phase (P), CNCA can inform risk management, strategy and resource allocation decisions (P1), setting and managing performance against targets (P2) and preparing reporting and disclosures (P3) (figure 4).

Forico found that their CNCA was especially useful for informing the Evaluate and Prepare phases of the TNFD LEAP approach [43]. As the Forico example shows, NCA can provide businesses with data and information that can be useful for business applications including but not limited to nature-related disclosures. NCA provides a systematic process of identifying, measuring, recording, summarizing and reporting consistent data and statistics that can inform assessments of nature-based risks and opportunities in a consistent, repeatable way over time.

## 5. The role of NCA in supporting business applications

Businesses can benefit from the structure, concepts, and the data and information provided by NCA to inform management decisions, reporting and disclosures related to nature. While this paper focuses on the TNFD as a tangible example, there are other emerging examples where NCA can also be useful.

Both NNCA and CNCA can provide integrated datasets that can be used for analyses required to understand and inform management of nature-related risks and opportunities for business. NNCA could be a useful source of credible data and information if available in the country(ies) of relevance for businesses that are developing CNCA. This is particularly true when it comes to tracking trends consistently across time, space and sectors. For example, SEEA Central Framework water accounts can provide consistent, structured data describing watershed-scale trends in water use and water quality [45], while SEEA Ecosystem Accounting data can track changes in related ecosystem services such as the regulation of sediment retention [46,47]. These data are often not measured or monitored by individual businesses, yet could be useful for informing corporate risk analysis related to ecosystem conditions upon which many different businesses, sectors and/or communities may depend. For example, in Australia, ecosystem accounts have been helpful for identifying the impacts of catchment management on water availability and have highlighted risks to a water supply company [48].

Because NNCA can provide data and information that can be used at multiple scales and by numerous sectors and businesses within a region, it provides an opportunity for many different actors to use the same integrated datasets and underlying models when developing individual CNCAs to inform business decision-making. For this reason, NNCA data can support comparability and benchmarking with respect to business's positive and negative impacts on shared, or communal, natural resources and ecosystem services within a landscape. In addition, tools such as the U.N. Global Platform and ARIES (box 1) could supply information for both NNCA and CNCA, which would help ensure that the data and approaches used for both are consistent across sectors and within ecosystems where multiple companies source, operate and have impacts and dependencies. In the Forico case, the company uses government data and approaches for watershed modelling that provide outputs that are integrated into their CNCA, which helps promote informational consistency when describing the state of the watershed that Forico, other businesses and a range of stakeholders depend upon and impact.

Because SEEA is a recognized global statistical standard, the data and information generated from SEEA-aligned NNCA can help increase credibility of natural capital information used by businesses such as Forico for CNCA or other business applications. The credibility of SEEA can also support assurance of nature-related data and information due to the limited number of accounting professionals who are familiar with assessing the quality and credibility of nature-related data, metrics and measurement approaches.

## 6. Strengthening NNCA and CNCA together: toward robust cross-sector accounting for nature

As NNCA advances and countries commit to including natural capital in official economic statistics, it is important to consider both how NNCA can be developed to better support business decisions and key applications such as reporting in alignment with the TNFD, as well as the role that business could play in supporting a robust NNCA system. Businesses regularly use national economic accounts data to forecast the macroeconomic business environment; NNCA could similarly be used by businesses in the future. Three recognized challenges for improving the relevance of NNCA for business include: (i) prioritizing the development of business-relevant accounts and integrated datasets (with final data disseminated alongside accompanying summary data and tools capable of generating custom queries); (ii) addressing latency issues in NNCA;^4^ and (iii) ensuring NNCA data and information can be used at granular spatial scales that are relevant to business dependencies and impacts on nature [14].

Certain types of nature-related data will be easier for businesses to leverage from NNCA than to collect on their own. For example, many companies may already track information on certain nature-related impacts, such as their water consumption and discharge metrics. However, information on basin-wide water use and water quality trends, and land use, land cover and ecosystem condition (or integrity) trends that may influence water quality and water flows in locations where businesses have operations or supply chains, may not be tracked by individual businesses, but could be provided by NNCA due to the multiple spatial scales at which NNCAs track, organize and present data [49]. Businesses may not be aware of or collect data on dependencies or impacts on nature that are tied to ecosystems or ecological processes that deliver benefits across spatial scales much greater than their own operational or supply chain footprints. For example, many businesses may not collect data or track information on critical ecosystem services, such as water filtration, flood and storm mitigation, wild pollination and/or pest control [50].^5^ Yet, measuring and managing such services effectively are important for business and society given they comprise a significant proportion of the economic value of an ecosystem [52] and are critical for all economic activities [2]. In such cases, SEEA-aligned NNCA, which follows consistent standards for tracking natural capital data across industries and at multiple spatial scales [47,49], could be especially helpful for supporting integrative business applications such as CNCA and TNFD-aligned risk management and reporting.

**Figure 5.**
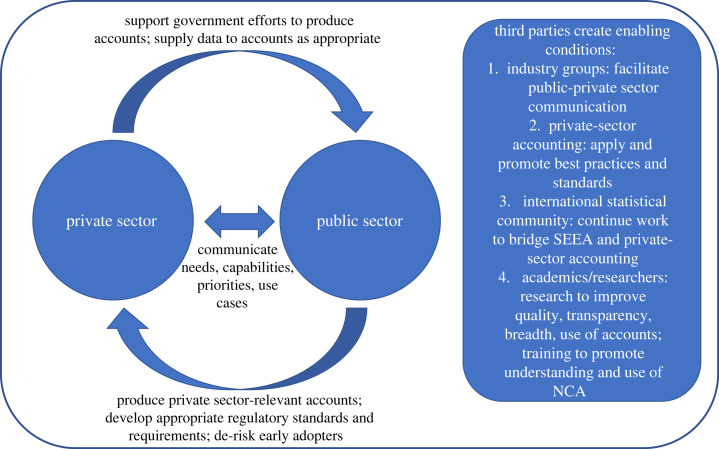
The virtuous circle of interactions between the public and private sector regarding the use and benefits of natural capital accounting (NCA) for business.

Ideally, business data and reporting on drivers of nature impacts, similar to other reported environmental impacts such as air emissions and water effluents, could also be a source of data to inform NNCA [14]. CNCA as defined by the Capitals Coalition [10] can support such possibilities by promoting data standardization, providing governments with another reason to promote the use and maintenance of NNCA. In addition to providing data, opportunities exist for businesses to support government-led NCA efforts by demonstrating use cases for the accounts and exchanging insights and experiences on practices in measuring and valuing nature for decision-making. National governments play several roles related to the wider use of CNCA—through their statistical role, producing and making available relevant NNCA information, and—through their regulatory role in developing appropriate standards and requirements—they may help to de-risk early adoption of CNCA by businesses. Additionally, third parties—including industry groups, private-sector accountants, audit and assurance professionals, the international statistical community and academics—can also support enabling conditions for a ‘virtuous circle’ that simultaneously advances both NNCA and CNCA (figure 5).

## 7. Addressing practice barriers to adoption of natural capital accounting to support business decisions

By addressing seven key areas, the awareness and adoption of NNCA and CNCA to support nature-related business measurement, assessments and reporting aligned with frameworks such as TNFD could be substantially improved, as described below.

### (a) Data availability and access, including data provenance and country sovereignty

In order to be useful for CNCA, NCA data should be publicly accessible [6]. In some countries such as the USA, Australia, and the European Union, NNCA outputs are made publicly accessible. However, not all national statistical offices will have the capacity to do so. Resources like the U.N. Global Platform ([38], box 1) and/or proposed TNFD Global Nature-related Public Data Facility could provide greater capacity to make NCA data accessible. To do this, issues related to trust, power asymmetries and country data sovereignty may need to be resolved. Available supporting documentation, or metadata, should clearly describe the provenance of the data in order to maintain credibility. Finally, while open data are desirable in most cases, any approach to managing statistical data, including all forms of NCA, must be capable of compliance with legal and institutional confidentiality needs.

### (b) Data alignment at temporal scales of business needs

NNCA data are useful for businesses when they align with the temporal scale of business decisions, which vary by industry. For example, corporate reporting occurs at least on an annual basis in alignment with financial reporting time frames and external reporting rhythms, meaning that NNCA data would ideally be produced annually; however, they are currently typically produced far less frequently. For example, Forico has committed to publishing their natural capital accounts annually, and the TNFD's recommendations are on an annual reporting cycle basis. The nature-related data needed to support corporate reports should be updated regularly as needed, which may be less frequently than annual reporting cycles but more frequently than the national datasets used for many NNCA currently. As an example of this challenge, pilot ecosystem accounts for the Southeastern United States that were published in 2020 included a time series covering the years 2001–2011 [47]—a 9-year gap from 2011 to 2020 at the time of release that was due to unavailability of more recent data. Updated ecosystem accounts extending through 2019 were released in February 2023, reducing this gap to 4 years [53]. While the information included in these accounts, such as the extent of native pollinator habitat, air quality regulation, water purification and carbon storage, has relevance for a range of businesses, such temporal gaps significantly limit the use of such NCA information for decision-making, especially in the business context [14]. In the USA, work is underway to substantially reduce the latency of land cover data that underlie various models of ecosystem extent and services [54].

### (c) Data alignment at spatial scales of business needs

As emphasized by the LEAP approach, natural capital is spatially specific and yet many businesses are not aware of where dependencies or impacts on nature occur across their supply chains. Oftentimes, such impacts may occur far from where operations take place, such as downstream impacts from fertilizer application on upstream fields or forest degradation/loss that can affect the regulation of water flows and water quality many hundreds of kilometres downstream [55]. By explicitly including locational information, spatial data underlying NNCA can enable companies to better assess and manage nature-related risks across boundaries and jurisdictions, which may extend beyond the range and scale of their typical data collection, monitoring and measurement efforts.

While many businesses do not fully understand all of their impacts on nature, fewer are aware of all of their dependencies on nature outside of direct material inputs [5]. Many of the less understood business dependencies on nature may include reliance on ecosystem services that are generated over large spatial scales [56]. Because of the large spatial scales over which some ecosystem services, such as flood risk reduction, are generated and/or the complexity of quantifying such services, many businesses do not collect data related to such flows nor calculate associated financial risk exposure should these benefits be lost. As more companies begin to understand and address a broader range of ecosystem services, it may be useful to utilize common models and sources of data across NNCA and CNCA to ensure comparability across spatial scales, and sectors and companies that impact and depend on ecosystems and the services they support in the same locations. This approach was applied in the case of Forico, which used and applied water models that are also used by the government. Such consistency across business and government approaches may build confidence in businesses and investors that credible, consistent models and data have been used to estimate dependencies or impacts on shared ecosystems and the services they provide.

### (d) Corporate capabilities and technical needs for using NCA data

In countries where NNCA data are available, at least three approaches exist for business to access and use the data—(i) downloading, interpreting and using properly documented data produced by countries, (ii) using tools to access existing NNCA data (e.g. box 1) or (iii) generating integrated datasets for business applications. Downloading and interpreting NNCA data from a public data repository requires expertise in Geographic Information Systems and NCA to manipulate and interpret data correctly, which most businesses lack in-house. Many existing tools (e.g. [57]) require even greater expertise, such as scientific modelling skills needed to run and customize outputs to improve their credibility. Consultants and partners can fill some of these capacity gaps, although it is important that business leaders understand how to use and interpret nature data. Further, data and software licensing requirements must be taken into account, as some datasets, models and software tools either prohibit use by for-profit entities or require paid subscriptions for their use. The growth of nature data and analytics tools available for businesses may help address some of these data access challenges [9].

### (e) Accounting, audit and assurance profession capability considerations

In order to audit the statistics, disclosures and/or statements that NNCA or CNCA may inform, accounting, audit and assurance professionals who understand nature will be needed. Currently, many accounting firms provide non-financial assurance over corporate environmental, social and governance (ESG) claims and sustainability reports. However, given the specialized knowledge needed for interpreting the often complex results of many ecosystem models and nature data and metrics, many accounting, audit and assurance professionals will need formal training and specialized expertise and skills related to understanding and interpreting them effectively. Thus, audits related to nature data and metrics will need to continue to rely on experts such as ecologists, climate scientists and hydrological modellers, for example, as they currently do for other financial disclosures that may require experts who specialize in estimates, actuarial advice and valuations. Notably, CNCA establishes an audit trail that allows for auditing to be conducted of nature-based data and information. CNCA also calls for double-entry bookkeeping similar to the International Financial Reporting Standards.

### (f) De-risking: an additional key role for governments

Due to data gaps, data accessibility and usability, and technical capacity challenges addressed throughout this paper, it may not be possible for many businesses to assess all of their material nature-related dependencies and impacts and then accurately analyse how these create business risks and opportunities. If a business's estimates are wrong, they could face challenges from their stakeholders. Through the establishment and management of publicly accessible, repeatable, auditable and credible low-risk NNCA data, governments could play an important role in partially de-risking companies' CNCA datasets and other information used for assessments and reporting in alignment with frameworks such as the TNFD.

### (g) Understanding NCA-related costs and incentives for businesses

Corporate financial accounting is driven largely by mandatory standards required of corporations to publicly release financial accounting audits and reports. In the context of CNCA and other nature-related reporting and disclosures, both mandatory requirements, such as the European Sustainability Reporting Standards and the EU Regulation on Deforestation free products, and voluntary reporting frameworks such as the TNFD are driving business actions. In addition, investors are beginning to request this information from companies in an effort to reduce their financial risks associated with nature loss and take advantage of financial opportunities that can emanate from nature conservation and restoration. A global data platform could offer businesses opportunities to access natural capital information by lowering the costs associated with data collection, management, analysis and reporting, although cost savings will depend on factors such as governance, quality and interoperability with existing initiatives (box 1). Ideally, NCA will help businesses manage costs and generate benefits by identifying financial risks associated with nature loss and opportunities to create value from regenerative business practices, nature conservation and restoration actions that are compatible with local-scale economic development.

## 8. Conclusion

An increased understanding and use of SEEA-aligned NNCA can support the development of CNCA and can inform other business applications, such as TNFD reporting and disclosures. Both NNCA and CNCA can provide credible, regular, consistent and structured data that can support business efforts to understand, manage and report nature-related dependencies and impacts, and disclose nature-related risks and opportunities aligned with the TNFD. Collectively, all of this information should enable businesses to sustainably manage nature to support biodiversity, business, economic and societal outcomes. However, to maximize the potential for this, NNCA datasets need to be sufficiently granular, regularly updated and publicly available. Because SEEA is a statistical standard, NNCA-derived data and statistics used to assess and manage businesses' nature-related risks may also increase investor confidence in the data and regulators’ confidence when establishing nature-related standards.

In summary, NNCA can inform and support CNCA and other business applications, including TNFD-aligned analyses and reporting for risk management, by providing accessible, high-quality, credible information for aspects of natural capital that a company may not be tracking currently or able to track on their own. Both NNCA and CNCA provide a structure for organization and management of data that can be used by businesses to understand, track and manage nature-related risks and opportunities over time. Businesses can also help create the demand for and use of NNCA by encouraging governments to collect and report on data that would also be useful for their own CNCA and business decisions, collecting and sharing their own CNCA data where feasible, and using NNCA and self-collected data for assessing and reporting on their nature-related risks and opportunities. This will help inform an enabling environment and economy that drives investments into nature-based solutions and nature-positive outcomes, which are greatly needed to address global nature and biodiversity-related changes [58–60].

## Data Availability

This article has no additional data.

## Endnotes

1 The Align project ‘Aligning Accounting Approaches for Nature’ will assist the European Commission's efforts to support businesses, financial institutions and other stakeholders in developing standardized natural capital accounting practices by establishing a standardized approach to biodiversity measurement and valuation.

2 Transparent was an EU LIFE-funded project that developed standardized natural capital accounting and valuation principles for business in line with the ambition of the European Green Deal.

3 Other CNCA standards that provide consistent organizational structure include the British Standard for Natural Capital Accounting [32] and a parallel standard under development by the International Organization for Standardization [33].

4 Latency refers to the time elapsed between collection of data and their release. All else being equal, low-latency data, which are released closer to real-time (i.e. in a manner of months), are preferable for CNCA and NNCA applications than data with multiyear latency.

5 However, as a counter-example, businesses do track the influence of heat, which can affect the labour supply [51].
